# Effects of Behaviour Change Communication on Knowledge and Prevention of Malaria Among Women in Ghana

**DOI:** 10.1177/0193841X231194565

**Published:** 2023-08-11

**Authors:** Emmanuel Orkoh, Uchenna Efobi

**Affiliations:** 1 School of Economic Sciences, 56405 North-West University (NWU), Potchefstroom, South Africa; 2 Network for Economic Research and Technical Solutions (NERTS), Geneva, Switzerland; 3 Initiative for Policy, Evaluation and Research, Abuja, Nigeria

**Keywords:** behaviour change communication, malaria, propensity score matching, multidimensional index, Ghana, I10, I12, I18

## Abstract

Behaviour change communication (BCC) remains a central component of the interventions used in the fight against malaria in Ghana. However, there is limited evidence of its effectiveness. This study evaluated the effects of BCC strategies on knowledge (symptoms, causes and prevention) and overall knowledge of malaria among Ghanaian women aged 15–49 years. The propensity score matching (PSM) approach and logistic regression were used to analyse data from the 2016 edition of the Malaria Indicator Survey (MIS). Women who participated in community-level education or heard/saw media messages on malaria, or both, had significantly more knowledge of the disease than women who lacked access to any of these mediums of communication. The effect of these strategies on women’s overall knowledge of malaria is about 2% to 4% and is higher on their knowledge of the symptoms (3% to 6%) and prevention (2% to 4%) than the causes (2%). The combined effects of both mediums of communication are relatively higher than the effect of either of them as a single medium of communication. Further analysis showed that improved knowledge of the disease is associated with higher preventive measures taken by women for themselves and for their children. The results are more significant in rural and poor households than in urban and non-poor households. These findings underscore the need for the Ministry of Health and its partner institutions to adopt an innovative approach which combines the two strategies in intensively educating Ghanaians, and women in particular, on the symptoms and prevention of malaria, giving due cognisance to households’ socioeconomic status and geographical location.

## Introduction

Malaria remains a source of significant economic and social costs to societies in Africa, despite efforts by governments, civil society organisations and non-governmental organisations over the years to prevent and/or reduce the impacts of the disease. Among the many interventions rolled out to contain the disease are the distribution of insecticide-treated nets (ITNs), a shift from targeted ITNs to universal coverage with long-lasting ITNs (LLINs), regular indoor residual spraying, home management of malaria (HMM) and the implementation of the Affordable Medicines Facility–malaria (AMFm) (Quakyi et al., 2017). The World Health Organization (WHO) and its allied institutions are committed to reducing malaria-induced morbidity and mortality by 90% by 2030 and completely eradicating the disease by 2050. These ambitious objectives call for strategies that will shape effective policymaking and proper application of the various interventions (The Health Communication Capacity Collaborative (HC3), 2017).

A key component of the various interventions used to fight malaria is behaviour change communication (BCC), which refers to the strategic use of communications to encourage individuals and communities to adopt healthier and more sustainable practices (WHO, 2019). In 2012, Roll Back Malaria Partnership (RBM) introduced the Strategic Framework for Malaria Communication, which outlined clear priorities for strengthening countries’ capacity, improving programme strategies and sharing best practices of evidence-based communication to enhance the control and/or eradication of malaria (Whittaker et al., 2014).

BCC strategies are underpinned by behaviour change theories which help us to understand people’s actions and why their behaviours change. The application of the theories depends on the socioecological level in question: (1) individual (health belief model, theory of planned behaviour and stages of change [transtheoretical model]); (2) interpersonal (social learning theory); and (3) community (diffusion of innovation theory) (Nabavi, 2012). While each of these theories is relevant to an aspect of BCC, the one that is most applicable to the present study is the social learning theory. This theory posits that we learn from our interactions with others in a social context by observing their behaviours and developing similar behaviours through assimilation and imitation – particularly where our experiences are positive or there are rewards attached to our observed behaviour (Nabavi, 2012).

The practical application of the social learning theory requires the learner to observe and imitate the behaviour of others, see how positive behaviours are modelled and practised, increase their capability and confidence to implement new skills, and win support from their environment to implement those skills (Le & Hancer, 2021). The central principle of the theory is that behaviour is determined by environmental factors (social norms, access within the community and influence of others [ability to change own environment]), behavioural factors (skills, practice and self-efficiency) and cognitive or personal factors (knowledge, expectation and attitude) (Zentall, 2022).

The social learning theory has also been the basis of many health interventions, including malaria prevention in Ghana. The Ministry of Health (MOH), Ghana Health Service (GHS) and National Malaria Control Programme (NMCP), together with their key stakeholders, introduced the Ghana Malaria Strategic Plan (2008–2015) in 2007. A revised version was subsequently introduced covering the period 2010–2015. The strategic plan was intended to guide the development, implementation and monitoring of the BCC component of the malaria prevention and control process in the country . It specifically set out to (1) define communication and behaviour change objectives; (2) identify the key target groups, messages, channels and communication interventions that focus on awareness creation, as well as the key determinants of preventive and care-seeking behaviour that will expand the use of proven prevention and control interventions (MOH, 2010).

The effectiveness of similar BCC interventions in other developing countries, including Myanmar, Nigeria, Uganda, Tanzania, Cameroon and Zambia, has been evaluated in various studies (Boulay et al., 2014; Helinski et al., 2015; Nyunt et al., 2015; Zalisk et al., 2019). However, such studies focused on one aspect of malaria at a time: symptom, causes or prevention. While those particular studies found BCC interventions to be effective, other studies suggested that simply increasing knowledge and awareness of a phenomenon such as malaria does not necessarily translate into behaviour change. Instead, behavioural and sociocultural factors (generally classified as social determinants) are relevant when determining behaviour change (Lamstein et al., 2014; Nair et al., 2016). Koenker et al. (2014), in turn, argue that although there is a rising number of studies examining the effectiveness of malaria BCC, more high-quality data is needed, especially as transmission dynamics change.

**Figure 1.**
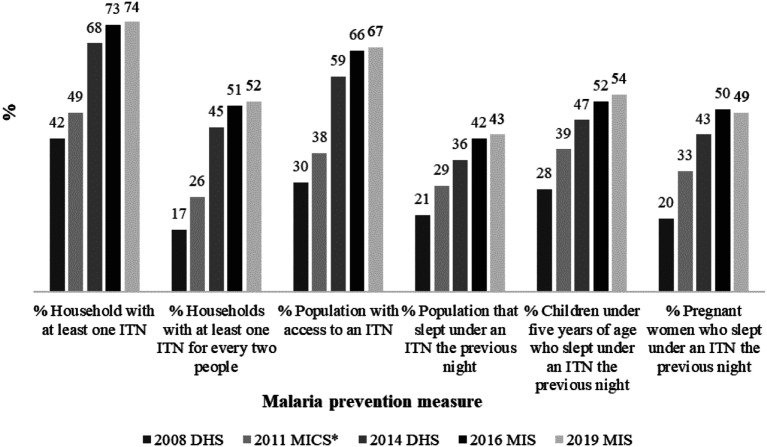
Trends of ITN ownership and utilisation (2008–2019). Source: Authors’ computation based on information from PMI (2022).

In the Ghanaian context, available data (see Figure 1)^1^ from the President’s Malaria Initiative (PMI) (2022) suggest an improvement in malaria prevention measures since the implementation of the BCC in 2008. There were encouraging trends, both in terms of ownership of and use of ITNs, between 2008 and 2019. Although the data used to compute Figure 1 were extracted from different health surveys, the proportion of households with at least one ITN for two people almost quadrupled while the share of the population with access to ITNs more than doubled. Also, all the indicators of ITN utilisation more than doubled.

Studies that have empirically assessed the effectiveness of BCC, either in building knowledge of or preventing malaria in Ghana, remain patchy and are in some cases limited to the community or local level (Quakyi et al., 2017; Tweneboah-Koduah et al., 2012). In their evaluation of the effectiveness of the AMFm activities on malaria among targeted groups in the Asante-Akim North and South Districts of Ghana, Quakyi et al. (2017) observed that those who had received the intervention were more knowledgeable about how to correctly administer the malaria drug to children under the age of five years than those who had not been exposed to the intervention. A similar study by Tweneboah-Koduah et al. (2012) on the effects of various ITNs among pregnant women and children in Ghana showed that most of the interventions had focused on ITN acquisition, not the integration of messages about malaria prevention.

Owusu Adjah and Panayiotou (2014) used the 2008 Ghana Demographic and Health Survey (GDHS) data to assess the effects of malaria-related messages on ITN use for malaria prevention among children in Ghana. The study revealed that individual messages conveyed by a health worker or via a dedicated radio programme boosted the likelihood of one or more children sleeping under a net, compared to the night before. Using the same GDHS data to examine the relationship between household heads’ exposure to media messages and ITN use among children under the age of five years, Apo et al. (2015) arrived at a similar conclusion. They emphasised the need for community-based educational campaigns involving health workers to work towards the universal use of ITNs for children under the age of five years in Ghana.

While these studies suggest that the BCC campaign in Ghana has been successful, Tweneboah-Koduah et al. (2012) assert that entrenched sociocultural practices remain an obstacle to the effective application of the knowledge acquired through such interventions. Other factors impacting the effectiveness of the campaign are inadequate financial and human resources, poor access to roads and extreme poverty. For instance, Tweneboah-Koduah et al. (2012) cite instances in which pregnant women in some communities kept their nets until their babies were born. In addition to these challenges, most of the empirical studies on the BCC campaign have focused on how it has impacted the use of ITNs to prevent malaria. There is limited evidence of how the campaign has improved people’s knowledge of the disease.

The present study addressed this gap and the challenges associated with the BCC campaign, with two main objectives: it assessed the effect of the mediums of communication on women’s knowledge of malaria in Ghana and it analysed the effect of women’s acquired knowledge on the measures that they take to protect themselves and their children against malaria.

Regarding the first objective, three research hypotheses were tested: (1) women who participate in community-level education programmes on malaria have statistically more significant knowledge of the disease than those who do not participate in such education programmes; (2) women who are exposed to media messages about malaria have statistically more significant knowledge of the disease than those who are not exposed to such media messages; and (3) women who are both exposed to media messages and participate in community-level education programmes on malaria have statistically more knowledge of the disease than those who do not participate in community-level education or are exposed to media messages.

Regarding the second objective, two research hypotheses were tested: (1) women who have more knowledge of malaria are more likely to sleep under bed nets; (2) women who have more knowledge of malaria are also more likely to ensure that their children under the age of five years sleep under bed nets. Earlier studies have found some rural–urban and poor–non-poor differences in access to health information and utilisation of health care services. This suggests that the BCC campaign on malaria may be targeted at a particular segment of the population, such as the poor and rural residents, who are predisposed to malaria (Mandal, 2022). To investigate the differences in the effect of women’s exposure to the mediums of communication on their knowledge of malaria and the likelihood of their taking preventive measures, we disaggregated the analyses by households’ poverty status and geographical location.

The approach used in this study to address the hypotheses and arrive at the findings may be equally applicable to education campaigns relating to other diseases, including tuberculosis and diabetes, which are among the top 10 causes of death in Ghana.

The method used to address the study objectives is discussed in the next section, followed by a description of the data and sampling design. The results and discussion are then presented. The final section concludes this paper with some policy recommendations.

### Conceptual Framework

**Figure 2.**
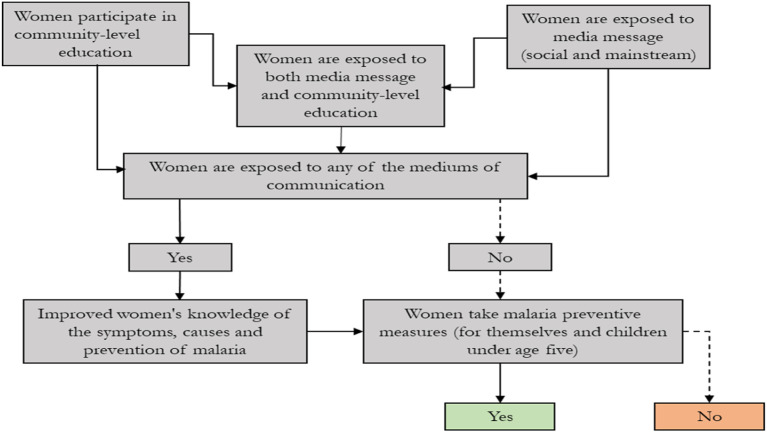
A framework of mediums of communication, knowledge and prevention of malaria. Source: Authors’ computation.

In support of the objectives and hypotheses outlined in the Introduction, Figure 2 presents the conceptual framework that links the mediums of communication to the knowledge and prevention of malaria. The figure shows that a woman may be exposed to the mediums of communication (community-level education, media messages about malaria prevention, or both) or may not be exposed to them.

Women’s exposure to any of the mediums of communication improves their level of knowledge of malaria and eventually enables them to take preventive measures for themselves and their family members against the disease. However, knowledge of the disease and the tendency to take preventive measures may be higher among women who are exposed to both mediums of communication than among women who are exposed to one or the other. It is also possible that a woman who is exposed to one or more of the mediums of communication may not take any preventive measures against the disease.

The right side of the conceptual framework (Figure 2) shows that women who may not be exposed to any of the mediums of communication may or may not take preventive measures, depending on factors such as the tendency to learn from others. Some of those women may still be influenced to take preventive measures against malaria if they live in a close neighbourhood and interact with women who take preventive measures against the disease due to their exposure to mediums of communication.

It can be inferred from the figure that exposure to the mediums of communication may have a positive spillover effect, which may translate into a positive externality as far as episodes of malaria in the close neighbourhood are concerned. The other side of the argument is that women who do not take any preventive measures may create some form of negative externality vis-à-vis the members of their communities. The externality may emanate from the cost (money and time) that other members of the household incur in taking care of these women who may be affected by malaria.

## Methods and Data

### Data and Sampling Design

This study relied on data from the 2016 Malaria Indicator Survey, which was jointly conducted across the 10 administrative regions by the Government of Ghana, the Ghana Statistical Service (GSS), the Ghana National Malaria Control Programme, the National Public Health Reference Laboratory (NPHRL) of the Ghana Health Service (GHS), and other international agencies, including the United States Agency for International Development (USAID) and the Global Fund. The sample frame used to identify the target households was based on the 2010 Population and Housing Census, which contains a complete list of all enumeration areas (EAs) in the 10 administrative regions of the country. An enumeration area covers an average of 114 households in rural areas and 185 households in urban areas (GSS et al., 2017).

The sampling process in the survey comprised two stages. The first stage involved the selection of EAs (93 in urban areas and 107 in rural areas), with the probability proportional to the size of the EA. A household listing operation was carried out in each EA from July to August 2016, and the list was used as a sampling frame. The second stage involved the random selection of 30 households from each EA to make up a total sample size of 6,003 households (2,912 in urban areas and 3,091 in rural areas). Although 5,929 households were occupied at the time the fieldwork was conducted, 5,841 (2,876 in urban areas and 3,053 in rural areas) were successfully interviewed, generating a response rate of 98.5%. The survey was conducted without allowance being made for the replacement of the non-responding unit. However, sample weights were generated for each unit to account for differences in the response rate and the non-proportional allocation of the sample across the 10 regions (GSS et al., 2017).

All the women participating in the survey were between the ages of 15 and 49 and resided in the household at the time of the survey. Non-permanent residents of the selected household, such as visitors who stayed in the household the night before the survey was conducted, were considered eligible for interviews. In the 5,929 occupied households, 5,186 eligible women were identified for interviews, but 5,150 were eventually interviewed, translating into a response rate of 99.3%. Children in the household between the ages of 6 months and 49 months were tested for anaemia and malaria infection, with the consent of their parents/guardians.

The survey instrument was divided into three modules: the household responses, the woman responses and the biomarker, with each module addressing different topics. The module on woman responses gathered their basic sociodemographic information, reproductive history over the past five years, preventive malaria treatment used for their most recent birth, knowledge of malaria, and exposure to community-level education and media messages about malaria, among other topics. For the estimation, the study relied on the modules on household responses and woman responses. The final samples used for the analysis varied across the models for knowledge of malaria and preventive measures against the disease. The sample size of respondents with knowledge of malaria and medium of communication indicators was 3,235; however, the sample size of respondents who were exposed to both community-level education and media messages about malaria was 1,943.

As a result, the final sample (reported at the bottom of Tables 2 and 4, respectively) for the regression estimates, in which the respondents had exposure to media messages and participated in community-level education, was 3,235. However, the sample for the model in which exposure to both mediums of communication was the explanatory variable of interest was 1,943. The sample size for the model of malaria prevention measures for women was 3,235, while the sample size for the model of malaria prevention measures for children under the age of five years was 3,142. While these were the sample sizes for the variables of interest, the covariates in each of the models had varied observations, which reflected the differences in the sample sizes reported in each of the results tables.

### Instrumentation

We computed four dependent variables (indices) to assess the effect of mediums of communication on knowledge of malaria in Ghana. These variables were used to measure respondents’ knowledge of the symptoms, causes and prevention of the disease, with a combined index measuring all three subcomponents (overall knowledge). In respect of each subcomponent, the Ghana Statistical Service asked respondents a set of questions which constituted the basis for the computation of the index.

Respondents were asked 10 sets of binary-response questions (see Table 1) relating to their knowledge of the symptoms of malaria. The responses to these questions were recoded as ‘1’ (one) if a respondent provided the right answer or ‘0’ (zero) otherwise. Similarly, respondents were asked to respond to a set of 12 binary-response questions on their knowledge of the causes of malaria. The final set of 13 binary-response questions covered respondents’ knowledge of the prevention of malaria in the country.

**Table 1.** Items and Weights Used to Compute the Indices.

Symptoms of malaria (*N* = 3,235)		Causes of malaria (*N* = 3,235)		Prevention of malaria (*N* = 3,235)	
Items	Weight (1/3)	Items	Weight (1/3)	Items	Weight (1/3)
Feveract	1/10	Sweet	1/12	Use net	1/13
Diarrhoea	1/10	Sun	1/12	Use ITN	1/13
Dizziness	1/10	Contaminated food	1/12	Use repellent	1/13
Loss of appetite	1/10	Mosquito bite	1/12	Use mosquito spray	1/13
Weakness	1/10	P-falciparum	1/12	Clear weeds	1/13
Cough	1/10	Hereditary	1/12	Clear stagnated water	1/13
Chills	1/10	Surrounding	1/12	Clean surroundings	1/13
Bitterness	1/10	Weedy surrounding	1/12	Use mosquito screen	1/13
Urine colour	1/10	Stagnated water	1/12	Avoid outside	1/13
Eye colour	1/10	Eating oily food	1/12	Avoid cold food	1/13
		Housefly	1/12	Avoid contaminated food	1/13
		Hygiene	1/12	Hygiene	1/13
				Clothes	1/13

Source: Authors’ computation based on data from MIC 2016.

Following earlier studies on the computation of the multidimensional index (Alkire & Foster, 2011; Efobi & Orkoh, 2017), we assigned an equal weight of 1/10 to each of the 10 items on the symptoms of malaria. We followed the same procedure by assigning an equal weight of 1/12 to the 12 binary-response items on the causes of the disease and an equal weight of 1/13 to the 13 binary-response questions on knowledge of malaria prevention. Since the overall index is made up of these three components (symptoms, causes and prevention), we assigned a weight of 1/3 to each of the three components. The product of this weight and the weights of the individual variables were multiplied by the respective variables. The process followed to compute the subcomponents of the index can be functionally expressed as equation (1).

(1) $$\theta_{i}=\pi_{1}\chi_{1}+\pi_{2}\chi_{2}+\ldots +\pi_{k}\chi_{k}$$

where θ is the subcomponent (symptoms, causes or prevention) of the multidimensional index expressed as a function of the sum of the product of each variable i and its weight π . Each variable was recoded to take the value of 1 (one) if a respondent was able to provide the right response to a question on it and zero (0) otherwise. The sum of the weights is represented as $\sum_{i=1}^{k}\pi_{i}=1$ . In other words, equation (1) can be simplified as equation (2).

(2) $$\theta_{i}=\sum_{i=1}^{k}\pi_{i}\chi$$

The final stage involved the summation of the product of the weights of each subcomponent to arrive at the index of the overall knowledge of malaria, which can be functionally expressed as equation (3).

(3) $$\Gamma_{\alpha }=\phi_{1}\Upsilon_{1}+\phi_{2}\Upsilon_{2}+\ldots +\phi_{d}\Upsilon_{d}=\sum_{\alpha =1}^{d}\phi_{\alpha }\Upsilon$$

where Γ is the overall index of knowledge of malaria expressed as a function of the sum of the product of each subcomponent Υ and its weight ϕ (1/3). Each of these four indices which constitute the dependent variables ranges from 0 (no knowledge) to 1 (full knowledge). However, for ease of interpretation, each index is multiplied by 100.

Unlike previous studies that have focused on one medium of communication at a time, this study computed three independent variables (women’s participation in community-level education on malaria prevention and control, women’s exposure to media messages on malaria, and women’s exposure to both mediums of communication) which were used for the estimation of the treatment effects. Each of these independent variables used the control group as the reference factor level.

To assess the extent to which women’s knowledge of malaria influences the preventive measures that they take for themselves and their children who are under the age of five years, two dependent variables were considered. The first dependent variable was binary, taking the value of 1 if the woman slept under a bed net the night before the survey and 0 otherwise. The second variable was equally binary, taking the value of 1 if the woman’s child under the age of five years slept under a bed net the night before the survey and 0 otherwise. Table 2 provides a detailed description of each of the variables and their respective summary statistics.

**Table 2.** Summary Statistics of the Variables Include in the Models.

Variable	Measurement	Obs.(*N*)	Mean	SD	Min	Max
Community-level education	A binary variable (1 if respondent participated in any community-level education; 0 otherwise)	3,157	.065	.246	0	1
Media message	A binary variable (1 if respondent has seen/heard any malaria messages on the media in the past six month; 0 otherwise)	3,157	.427	.495	0	1
Community-level education and media message	A binary variable (1 if respondent has seen/heard media message and participated in community-level education on prevention and control of malaria; 0 otherwise	1,902	.078	.268	0	1
Level of education	A categorical variable (1 None; 2 basic education and 3 secondary plus)	3,157	1.172	.964	0	3
Ethnicity	A categorical variable (1 Akan; 2 Ga-Dangme; 3 Ewe; 4 Guan; 5 Mole-Dagbani; 6 Grusi; 7 Gurma; 8 Mande; 9 Other)	3,157	3.618	2.378	1	9
Wealth quintile	A categorical variable (1 poorest; 2 poorer; 3 middle; 4 richer; 5 richest)	3,157	2.515	1.424	1	5
Age	A continuous variable measuring the age of the respondent as of the time of the survey	3,157	30.251	6.827	15	49
Child’s age	A continuous variable measuring the age (in month) of the child	3,034	28.193	17.261	0	59
Children under age five	A continuous variable measuring capturing the number of children under age five in the household	3,235	1.875	1.053	0	6
Sex of child	A binary variable which takes the value 1 if the child is female and 0 if the child is male	3,235	.488	.500	0	1
Region of residence	A categorical variable (1 Western; 2 Central; 3 Greater Accra; 4 Volta; 5 Eastern; 6 Ashanti; 7 Brong Ahafo; 8 Northern; Upper East; 10 Upper West)	3,157	5.767	2.752	1	10
Geographical location	A binary variable (1 if respondent lives in urban area; 0 otherwise)	3,157	.382	.486	0	1
Symptoms of malaria	A continuous variable (index) ranging from 0 and 100	3,157	34.246	11.385	0	100
Causes of malaria	A continuous variable (index) ranging from 0 and 100	3,157	40.103	7.730	16.667	100
Malaria prevention	A continuous variable (index) ranging from 0 and 100	3,157	28.641	8.278	7.692	100
Overall knowledge	A continuous variable (index) ranging from 0 and 100	3,157	30.754	6.930	14	100
Mother slept under bed net	A binary variable (1 if respondent slept under bed net during night preceding the survey; 0 otherwise)	3,235	.583	.493	0	1
Child slept under bed net	A binary variable (1 if a child under 5 slept under bed net during night preceding the survey; 0 otherwise)	3,142	.624	.485	0	1

Source: Authors’ computation based on data from MIC 2016.

### Estimation Technique

This study used the propensity score matching (PSM) approach to assess the impact of BCC on respondents’ knowledge of malaria. Ideally, an assessment of this nature requires a pure randomised control trial (RCT) which is mostly designed to test a hypothesis under an optimal setting in the absence of confounding factors (Saturni et al., 2014). However, the same objective can be achieved using a quasi-experimental approach like the PSM for observational data gathered through a survey (Boulay et al., 2014).

The quasi-experimental approach involves the identification of a comparison (control) group that is as similar as possible to the treatment group in terms of baseline characteristics (White & Sabarwal, 2014). The comparison group in this study were the women who were not exposed to any of the mediums of communication. The difference in outcome between the treatment and control groups can be attributed to the intervention. One advantage of PSM is that it permits an adjustment for selection bias when assessing causal effects in observational studies (Biondi-Zoccai et al., 2011).

The estimation process uses a treatment effect model where a dummy variable indicating the treatment condition (‘1’ if a woman was exposed to any of the mediums of BCC and ‘0’ otherwise) is directly included in the regression equation. The outcome variables (index of symptoms, causes and prevention and the aggregate index of knowledge of malaria) of the regression equation are observed for both observations (0, 1) of the dummy (policy) variables.

The first step in computing the PSM involves the estimation of the predicted probabilities that a woman would be exposed to any of the three mediums of communication using logistic regression

(4) $$Prob\left({Med\_com}_{i}=1|X_{i}\right)=\beta_{0}+\beta_{1}{Age}_{i}+\beta_{2}{Edu}_{i}+\beta_{3}{Ethnic}_{i}+\beta_{4}{Poor}_{i}+\beta_{5}{Urban}_{i}+\beta_{6}{Rgeion}_{i}+\mu_{i}$$

In equation (4), ${Med\_com}_{i}$ represents the probability that a woman would be exposed to any of the mediums of communication conditioned on a vector of individual and household characteristics (age, level of education, ethnic affiliation, poverty status of the household, place of residence and regional location of the household). The variables $\beta_{0}$ , and $\mu_{i}$ represent the constant term and the error term. This study focused on three intervention variables of interest (exposure to media messages on malaria, participation in community-level education on malaria and exposure to both mediums of communication) and the computed indicator for exposure to each of the variables. Each variable was recoded to take the value of 1 if the response to the question was Yes and 0 if the response was No.

The next step in the estimation process involves the application of three algorithms (i.e. nearest-neighbour matching (NNM), kernel matching (KM) and the radius-matching (RM) technique) to obtain robust matching estimates. The statistical significance of the average treatment effects on the quantities treated was tested using bootstrapped standard errors, which accounts for the variation caused by the matching process. As presented in Table 1, the observable pre-treatment covariates used to identify similar individuals were respondents’ level of education, age, ethnic affiliation, household wealth status, geographical location, region of residence, radio and television.

The choice of the covariates was informed by two main conditions, as discussed in the literature (Caliendo & Kopeinig, 2008; Rosenbaum, 2002). The first condition is that only variables that simultaneously influence the treatment status (exposure to BCC) and the outcome variables (knowledge of malaria indicators) should be included in the model. The second condition is that the variables included in the model should not be confounded. The outcome variable(s) must be independent of the treatment conditional on the propensity score (Angrist & Kuersteiner, 2011).

We estimated the average treatment effects by specifying knowledge of malaria variables as functions of the three treatment variables (exposure to any of the mediums of communication) in equations (5)–(7).

(5) $$\varphi_{i}=E\left\{Y_{i1}-Y_{i0}|D_{i}=1\right\}$$

(6) $$=E\left\{E\left\{Y_{i1}-Y_{i0}|D_{i}=1,p\left(X_{i}\right)\right\}\right\}$$

(7) $$=E\{E\{Y_{i1}\left|D_{i}=1,P\left(X_{i}\right)-E\left\{Y_{i0}|D_{i}=0,p\left(X_{i}\right)\right\}\right|D_{i}=1\}$$

The subscript i represents a woman, φ is the average treatment effect, while D is the binary variable which takes the value of 1 if the respondent was exposed to any of the mediums of communication and 0 otherwise. The variable Y represents the outcome variable (index of knowledge of malaria). The propensity scores, $P\left(X_{i}\right)$ , capture the probability that a respondent would be exposed to any of the mediums of communication, given the covariates (*X*). To validate the consistency of the results, we used three matching techniques, that is, NNM, KM and RM.

The second objective of this study involved assessing the effect of improved knowledge of malaria on preventive measures that women take for themselves and their children under the age of five years. This objective was addressed using logistic regression. The preventive measures (discussed in the preceding subsection) are specified as functions of the individual and household characteristics.

(8) $$Prob\left({Prev}_{i}=1|M_{i}\right)=\alpha_{0}+\alpha_{1}{Knowledge\_mal}_{i}+\alpha_{2}{Age}_{i}+\alpha_{3}{Edu}_{i}+\alpha_{4}{Insurance}_{i}+\alpha_{5}{Poor}_{i}+\alpha_{6}{Ethnic}_{i}+\alpha_{7}{Urban}_{i}+\alpha_{8}{Region}_{i}+\epsilon_{i}$$

$$\alpha_{1}>0,\alpha_{2}>0or<0,\alpha_{3}>0,\alpha_{4}>0,\alpha_{5}>0,\alpha_{6}>0or<0,\alpha_{7}>0,\alpha_{8}>0or<0$$

The vector ${Prev}_{i}$ represents the probability that a woman i would take preventive measures for herself and/or her child under the age of five years condition on the vector $M_{i}$ which represents the explanatory variables on the right-hand side (knowledge of malaria indicators, age, level of education, ownership of insurance, poverty status of household, ethnic affiliation, place of residence or geographical location, and regional location of household). In addition to these variables, the child’s model contains his/her age, sex and the number of children under the age of five years in the household.

Consistent with the conceptual framework, it was expected that an improved knowledge of malaria would translate into a higher probability that a woman would take preventive measures for herself and her child under the age of five years. The literature suggests that people’s adoption of preventive health care behaviours is largely influenced by their perception of ageing and the socioeconomic condition of the household (Levy & Myers, 2004). While a strong perception of ageing is positively associated with preventive health care, the relationship between a mother’s age and the likelihood of her sleeping under bed nets is influenced by the affordability and availability of the bed nets, the age of the child and the household’s sleeping arrangement.

Mothers who do not sleep in the same room as their children under the age of five years but who have limited access to bed nets are likely to sacrifice their own health preventive measures for those of their children. Inversely, mothers who live in households that have access to bed nets are more likely to use them, even if they do not sleep in the same room as their children. In line with the principle of altruistic parenting (Doepke & Zilibotti, 2017), it is expected that age will be negatively associated with the likelihood that a mother would sleep under a bed net but positively associated with the likelihood that the mother would ensure that her child under the age of five years would sleep under a bed net.

Education improves people’s health prevention behaviours (Asmah & Orkoh, 2017). It is therefore expected that the mother’s level of education will be positively associated with the likelihood that both she and her child under the age of five years will sleep under a bed net. Ghana’s health insurance scheme is linked to the concept of free maternal healthcare. Mothers who attend prenatal and antenatal care receive ITNs from public agencies (agents), non-governmental organisations (NGOs) and community-based agents (CBAs), either freely or at a highly subsidised cost (Orkoh & Annim, 2017). Nonetheless, an earlier study found evidence of ex-ante moral hazard associated with the health insurance scheme, especially when the level of effort and costs required for prevention is high (Yilma et al., 2012). In this analysis, it was expected that ownership of health insurance would be positively associated with bed net utilisation because of the affordability of distribution.

Poverty is negatively associated with preventive health care. However, in the Ghanaian context, the majority of households obtain free or highly subsidised bed nets from health care centres and other government agencies on the grounds of greater affordability, access and equity. In this regard, it was expected that poverty would be positively associated with the use of bed net. Social norms associated with ethnicity render the latter’s relationship with preventive health care indeterminate. Some sociocultural practices do not encourage the adoption of health preventive measures like the use of bed nets. The relationship between ethnicity and bed net use was therefore indeterminate in this analysis.

Urban residents have access to information on malaria control, but the BCC policy targets rural residents and vulnerable households. On average, urban residents can afford alternative malaria preventive measures apart from bed nets. It was expected that urban residents would be negatively associated with the use of bed nets. Like the place of residence, the relationship between regional location of households and the use of bed nets is dependent on the level of development of the region in question. In this analysis, the relationship between the region of residence and the use of bed nets was indeterminate. It is important to note that since this study focused on knowledge of malaria prevention, the estimates of these covariates were not presented in the results and discussion section.

## Results and Discussion

**Figure 3.**
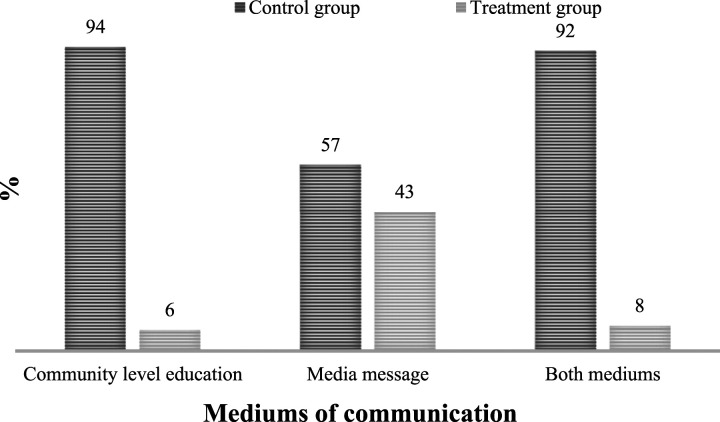
Women’s exposure to the mediums of communication. Source: Authors’ computation based on data from MIC 2016.

The discussion of the treatment effect estimates in this section is preceded by a brief descriptive analysis of the four indicators of knowledge of malaria across women’s exposure to the three mediums of communication. The distribution of women’s exposure to the mediums of communication (see Figure 3) indicates that 43% of them were exposed to media messages while only 6% participated in community-level education on malaria. Similarly, only 8% of women were exposed to both mediums of communication.

**Figure 4.**
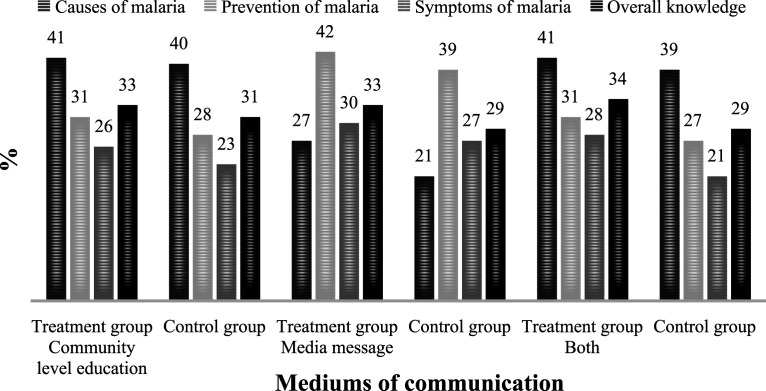
Knowledge of malaria by mediums of communication. Source: Authors’ computation based on data from MIC 2016.

Regarding knowledge of malaria, Figure 4 shows that women who were exposed to the mediums of communication were more knowledgeable than women who were not exposed to any of the mediums of communication. Knowledge of the causes, prevention and symptoms and overall knowledge of the disease was about one, five and three percentage points higher among women who participated in community-level education than among those who did not participate in such education. As far as media messages were concerned, the respective differences in knowledge of the disease were six, three and three percentage points. Regarding both mediums of communication, the respective differences in knowledge of the disease were three, four and three percentage points. These percentage point differences were reflected in the overall knowledge of the diseases.

We validated these results by running an independent group *t* test to assess whether women’s knowledge of malaria differed on the basis of their exposure, or otherwise, to the mediums of communication. The results (see Table 3) show that the differences in the means of knowledge of the symptoms and prevention of malaria between women who were involved in community-level education and those who were not exposed to such education are significantly different from 0 (zero). However, the result pertaining to knowledge of the causes of malaria is not significant. Similar significant differences are observed in the means of knowledge of women who were exposed to media messages and to both mediums of communication. There is also a statistically significant difference in the means of knowledge of women who were exposed to all three mediums of communication compared to those who were not exposed to any of the mediums of communication.

**Table 3.** Compare-Mean Tests of Knowledge of Malaria Indicators by Mediums of Communication.

Variable	Involved in community-level education (*N* = 3,157)				Exposed to media message (*N* = 3,157)				Exposed to both mediums (*N* = 1,902)			
	Total	Control	Treatment	Difference	Total	Control	Treatment	Difference	Total	Control	Treatment	Difference
Symptoms of malaria index	33.732 (.286)	33.443 (.295)	37.952 (1.103)	−4.508*** (1.165)	33.732 (.286)	30.240 (.355)	38.407 (.439)	−8.167*** (.559)	30.942 (.357)	30.176 (.364)	40.175 (1.383)	−9.999*** (1.323)
Causes of malaria index	60.547 (.207)	60.487 (.213)	61.435 (.835)	−.948 (.844)	60.547 (.207)	58.868 (.259)	62.795 (.327)	−3.927*** (.412)	59.092 (.255)	58.841 (.262)	62.107 (.995)	−3.266*** (.955)
Prevention of malaria index	41.172 (.214)	40.953 (.220)	44.382 (.847)	−3.429*** (.872)	41.172 (.214)	39.245 (.267)	43.753 (.338)	−4.509*** (.425)	39.640 (.263)	39.162 (.270)	45.389 (.983)	−6.227*** (.979)
Overall knowledge index	30.755 (.122)	30.23 (.126)	32.687 (.477)	−2.064*** (.497)	30.755 (.122)	29.132 (.154)	32.928 (.182)	−3.795*** (.237)	29.437 (.153)	29.092 (.157)	33.588 (.572)	−4.496*** (.568)

Source: Authors’ computation based on data from MIC 2016.

The above brief descriptive analysis gives an idea of the potential effect of the mediums of communication on women’s knowledge of malaria. However, it does not provide enough information on the extent of the impact of the intervention. This is discussed in the next subsection which presents the treatment effect estimates.

**Figure 5.**
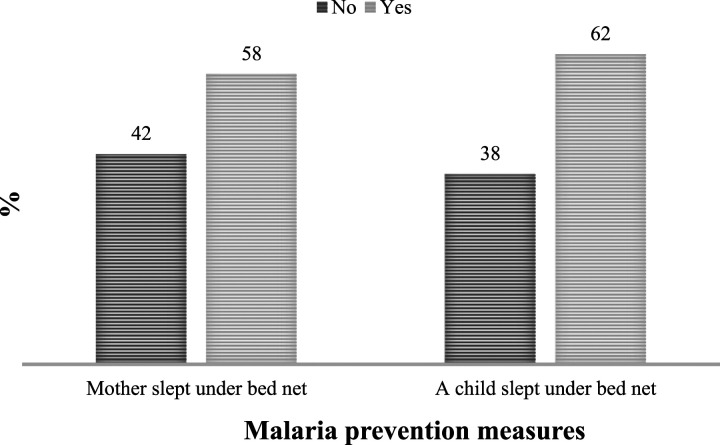
Use of bed net for malaria prevention among women and children. Source: Authors’ computation based on data from MIC 2016.

Regarding the preventive measures (see Figure 5), 42% of the 3,235 women who responded to the question on the use of bed nets did not sleep under mosquito nets during the night preceding the survey. Similarly, 38% of the 3,142 women questioned on whether a child slept under a mosquito net, indicated that the child had not slept under a mosquito net during the night preceding the survey. These statistics underscore the need for more educational campaigns to scale up the use of ITNs and other malaria-prevention measures in the country. As observed in the preceding paragraphs, low exposure to mediums of communication and limited knowledge of malaria among women with low levels of education call for a retargeting of the BCC campaign towards this segment of the population.

### Results of the Propensity Score

We begin the discussion of the treatment effect estimates with a description of the propensity score model which relates the covariates to the treatment (exposure to media messages or participation in community-level education on malaria). The results (see Table A1 in the Appendix) indicate that a woman who had a higher level of education (secondary plus) was more likely to be exposed to any of the three mediums of communication than a women who had no education. Women in wealthy households were also more likely to be exposed to the mediums of communication. As with education, women in wealthy households were more likely to have access to sources of information.

Aside from these factors, women’s exposure to any of the mediums of communication was largely dependent on the geographical location of their household. The estimates of geographical location (place of residence) indicate that a woman who lived in an urban area was less likely to be exposed to community-level education on malaria. This result is intuitive because community-level education on malaria in most developing countries like Ghana is predominantly a rural phenomenon. Those living in rural areas are more prone to the disease and have limited access to health care (Iqbal et al., 2016).

The analysis across the 10 regions of Ghana shows some heterogeneities of exposure, depending on the regional location of the household and the medium of communication. Compared to a woman in the Greater Accra region, a woman in the Central, Brong-Ahafo, Upper East or Upper West regions was more likely to participate in community-level education on malaria. However, a woman in the Western, Volta, Eastern or Ashanti region was less likely to be exposed to media messages on malaria. It can further be observed that, compared to a woman in the Greater Accra region, a woman in the Central, Brong-Ahafo, Upper East or Upper West was more likely to be exposed to both mediums of communication. As evidenced by the marginal effects, this finding is largely driven by the greater exposure of women in these regions to community-level education than to media messages.

**Figure A1.**
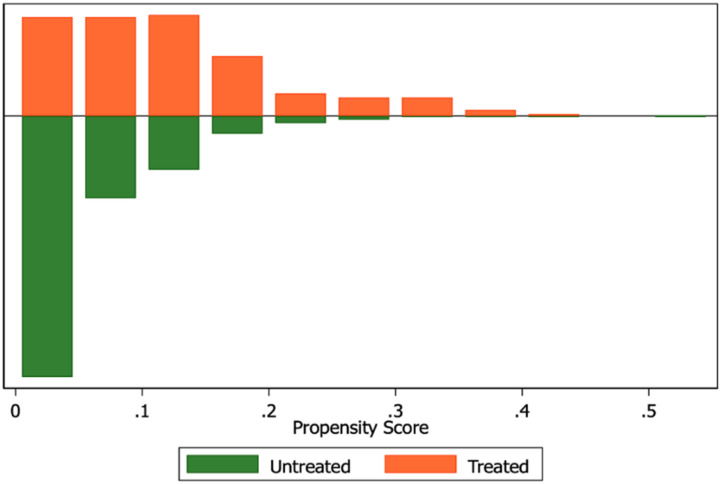
Distribution of the propensity scores – community-level education. Source: Authors’ computation based on data from MIC 2016.

**Figure A2.**
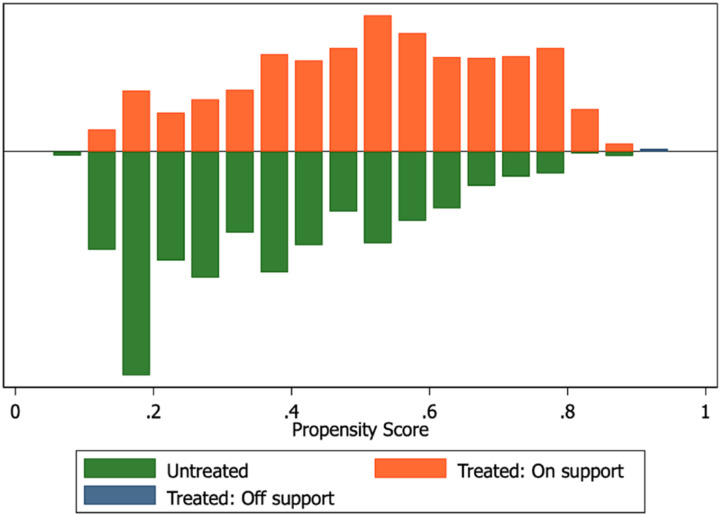
Distribution of the propensity scores – media message. Source: Authors’ computation based on data from MIC 2016.

**Figure A3.**
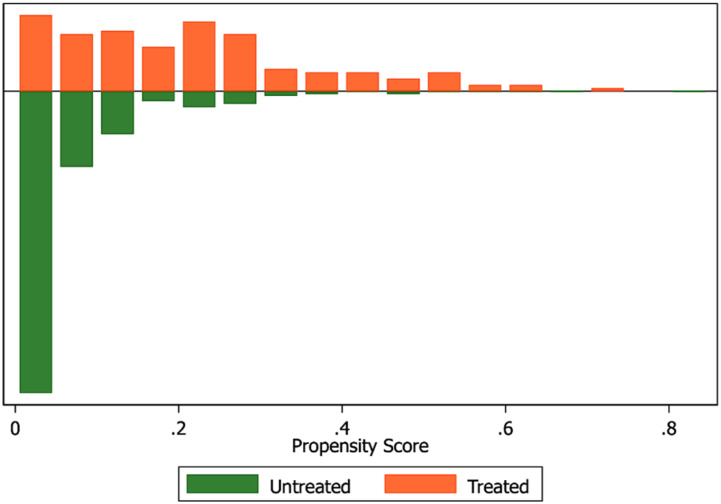
Distribution of the propensity scores – both mediums of communication. Source: Authors’ computation based on data from MIC 2016.

Figures A1–A3 in the Appendix evaluate the quality of the propensities matched for respondents who were exposed to a medium of communication and those who were not exposed to any of the mediums of communication. Figure A1 shows that all the covariates in the treatment group of the model for participation in community-level education were balanced after the matching. Thus, the observable individual and household characteristics used for calculating the propensities are sufficient for matching those in the treatment group (exposure to a medium of communication) and those in the control group (no exposure to a medium of communication). However, in the media message (see Figure A2 in the Appendix), only one of those in the treatment group was off support after the matching. Regarding exposure to both mediums of communication (see Figure A3), all the covariates were balanced after the matching.

Table 4 presents further checks of the matching quality by comparing the differences between the two groups of respondents – those who were exposed to the intervention and those who were not exposed to the intervention. The overall covariance distribution (mean and median absolute bias) and the model fit (pseudo R2 and LR-test) before and after the matching are reported in Table 4. The results for the NNM, KM and RM suggest that the pre-matching differences in the observable characteristics (e.g. mean absolute biases) of the sampled individuals across the two groups were significantly reduced after the matching.

**Table 4.** Matching Quality Checks.

Outcome Variable	Matching Algorithm	Sample	Pseudo $R^{2}$	LR $\chi^{2}$	${P>\chi }^{2}$	Mean Bias	Median Bias
Community education	Nearest neighbour (NNM)	Unmatched	.115	174.270	.000	16.300	13.700
		Matched	.013	7.610	1.000	3.500	2.600
	Kernel (KM)	Unmatched	.115	174.270	.000	16.300	13.700
		Matched	.231	12.970	.989	5.300	4.400
	Radius (RM)	Unmatched	.115	174.270	.000	16.300	13.700
		Matched	.139	22.260	.724	8.100	7.000
Media message	Nearest neighbour (NNM	Unmatched	.135	583.05	.000	16.400	9.700
		Matched	.005	17.16	.927	2.500	2.300
	Kernel (KM)	Unmatched	.135	583.05	.000	16.400	9.700
		Matched	.017	64.59	.211	5.300	5.000
	Radius (RM)	Unmatched	.135	583.05	.000	16.400	9.700
		Matched	.003	9.76	.999	1.900	1.700
Both mediums	Nearest neighbour (NNM)	Unmatched	.230	239.64	.000	21.900	20.700
		Matched	.014	5.840	1.000	4.000	2.800
	Kernel (KM)	Unmatched	.230	239.640	.000	21.900	20.700
		Matched	.037	14.670	.930	7.200	7.200
	Radius (RM)	Unmatched	.230	239.640	.000	21.900	20.700
		Matched	.018	7.4000	1.000	4.600	4.500

Source: Authors’ computation based on data from MIC 2016.

Consistent with the distribution of the propensity scores in Figures A1–A3, Table A2 in the Appendix shows that the observable individual and household characteristics used for the matching between the two groups have insignificant biases as far as participation in community-level education and exposure to both mediums of communication are concerned. In contrast, in the case of exposure to media messages, a couple of the categories of the characteristics had significant biases, but these biases do not necessarily discount the overall quality of the match.

### Treatment Effect Estimates

With the determinants of women’s exposure to the mediums of communication having been discussed above, this section discusses the treatment effect estimates. Separate estimation models were specified for the symptoms, causes, prevention and overall knowledge of the disease based on sub-sample analyses of respondents’ exposure to community-level education, media messages and a combination of the two mediums of communication. The results (see Table 5) generally show that the combined effects of media messages and participation in community-level education on malaria prevention are relatively stronger than the effect of either of them as a single medium of communication. The results further show that the effect of exposure to media messages is more significant than the effect of participation in community-level education on malaria prevention and control.

**Table 5.** Average Treatment Effect Estimates.

Dependent Variable	Community Education			Media Message			Both Mediums		
	NNM	KM	RM	NNM	KM	RM	NNM	KM	RM
Symptoms of malaria index	3.566*** (1.072)	1.565 (1.319)	3.588*** (.855)	3.022*** (.636)	2.478*** (.707)	2.951*** (.553)	6.260*** (1.315)	7.027*** (1.724)	6.090*** (1.109)
Causes of malaria index	.907 (1.166)	.208 (1.494)	1.033 (.851)	2.087*** (.573)	2.067*** (.727)	2.526*** (.496)	4.013*** (1.405)	3.557** (1.509)	3.417*** (1.099)
Prevention of malaria index	3.010* (1.544)	3.618** (1.641)	4.260*** (1.269)	6.573*** (.800)	5.678*** (.923)	6.494*** (.625)	10.550*** (1.965)	11.664*** (2.173)	9.549*** (1.530)
Overall knowledge index	1.947*** (.666)	2.554*** (.799)	2.012*** (.500)	2.822*** (.353)	2.505*** (.400)	2.738*** (.297)	4.273*** (.848)	4.537*** (1.047)	4.229*** (.624)
Observations	3,235	3,235	3,235	3,235	3,235	3,235	1,943	1,943	1,943

Standard errors in parentheses; ***p < .01, **p < .05, *p < .1. Source: Authors’ computation based on data from MIC 2016.

Consistent with the findings of previous studies (Apo et al., 2015; Owusu Adjah & Panayiotou, 2014; Tweneboah-Koduah et al., 2012), the results show that BCC is effective in improving the knowledge of respondents who are exposed to the mediums of communication. On average, a woman who participated in community-level education on malaria prevention and control was approximately 4% more knowledgeable on the symptoms of the disease than a woman who was not exposed to this medium of communication. The effect of exposure to media messages is around 3%, while the effect of both community-level education and media messages ranges from 6% to 7%.

Although the effect of women’s participation in community-level education on their knowledge of the causes of malaria is insignificant, the effects of exposure to media messages and both mediums of communication are approximately 2% and 4%, respectively. With regard to the effect of exposure to mediums of communication on knowledge of prevention of malaria, the effects of participation in community-level education range from 3% to 4%, while the effect of exposure to media messages is approximately 6%. The combined effects of the two mediums of communication range from 10% to 11%.

The estimates of the effect of the three mediums of communication on women’s overall knowledge (symptoms, causes and prevention) of malaria reveal that, on average, a woman who participated in community-level education on prevention and control of the disease was about 2% to 6% more knowledgeable than a woman who never participated in such education. In the same vein, a woman who was exposed to media messages on malaria had about 3% more overall knowledge of malaria (symptoms, causes and prevention) than a woman who was not exposed to this medium of communication. The effect of both mediums of communication on women’s overall knowledge of the disease is approximately 4%.

We further disaggregated the results by geographical location and poverty status of the household of the respondents to assess potential elements of segmentation in the malaria campaign with a view to targeting a specific population. The results (see Table 6) show that the extent of the effects and the level of statistical significance are higher among rural residents that among urban residents. In rural areas, a woman who participated in community-level education on malaria was about 4% more knowledgeable on the symptoms of the disease than a woman who did not participate.

**Table 6.** Treatment Effect by Household’s Location – Nearest Neighbour Matching (NNM).

Dependent Variables	Community-Level Education		Media Message		Both Mediums	
	Rural	Urban	Rural	Urban	Rural	Urban
Symptoms	3.897** (1.880)	2.719 (2.313)	7.657*** (.945)	5.598*** (1.211)	8.804*** (2.739)	5.762* (3.258)
Causes	2.851** (1.404)	−1.655 (2.069)	3.758*** (.739)	.116 (.971)	4.106** (1.727)	.740 (2.376)
Prevention	4.003*** (1.305)	4.216** (1.859)	3.538*** (.836)	2.407** (.956)	4.038** (1.604)	3.613 (2.465)
Overall knowledge	2.467*** (.792)	1.252 (1.049)	3.435*** (.468)	1.950*** (.541)	3.903*** (1.055)	2.378 (1.453)
Observations	1,860	1,235	2,000	1,235	1,213	525

Standard errors in parentheses; ***p < .01, **p < .05, *p < .1. Source: Authors’ computation based on data from MIC 2016.

The effects of exposure to media messages are statistically significant in both rural and urban areas although the extent of the effect is marginally higher among rural residents than among urban residents. A similar pattern of a stronger effect is observed in terms of other knowledge of malaria indicators. These results suggest that although rural residents may face structural barriers such as limited media exposure, which makes it harder for them to access health information and services (Chen et al., 2019), properly targeted BCC campaigns can have a stronger impact on their health outcomes.

Regarding the poverty status of the household (see Table 7), we found that the magnitude of the effects and the level of statistical significance were higher among respondents who lived in poor households than among those who lived in non-poor households. The effect of the BCC on knowledge of symptoms of malaria was higher than the effect on knowledge of prevention and causes of the disease. It is evident from these results that the BCC campaign has a statistically significant impact on the vulnerable segment of Ghana’s population. The results also support the conclusion of an earlier study that households’ economic conditions strongly influence knowledge of and efforts to prevent and treat malaria (Clouston et al., 2015).

**Table 7.** Treatment Effect by Household’s Poverty Status – Nearest Neighbour Matching (NNM).

Variable	Community-Level Education		Media Message		Both Mediums	
	Non-Poor	Poor	Non-Poor	Poor	Non-Poor	Poor
Symptoms	2.107 (2.098)	4.854** (1.968)	4.099*** (1.058)	8.566*** (1.005)	6.277** (2.903)	11.775*** (2.496)
Causes	.900 (1.908)	2.590* (1.392)	.088 (.826)	5.349*** (.689)	1.283 (2.234)	5.986*** (1.792)
Prevention	2.886 (2.026)	5.270*** (1.390)	.931 (1.002)	4.291*** (.745)	2.887 (2.445)	6.909*** (1.697)
Overall	1.007 (.945)	2.918*** (.838)	1.194** (.508)	4.177*** (.457)	1.842 (1.197)	5.683*** (1.000)
Observations	1,458	1,628	1,458	1,777	683	1,047

Standard errors in parentheses; ***p < .01, **p < .05, *p < .1. Source: Authors’ computation based on data from MIC 2016.

### Knowledge of and Preventive Measures Against Malaria

Table 8 presents the logistic estimates of the association between women’s knowledge of malaria and preventive measures that they take for themselves and their children under the age of five years. The results reveal that the prevention effect of knowledge of malaria among women is not limited to them alone; it also extends to their children under the age of five years. Controlling for other factors, a 1% increase in a woman’s knowledge of malaria prevention is associated with about a 9.2% probability that she would sleep under a bed net the night preceding the survey. The association between overall knowledge of malaria and a woman’s likelihood to use a bed net is about 1.5%, *ceteris paribus*.

**Table 8.** Knowledge of Malaria and Prevention Measures Taken by Women.

Variables	Mother Slept Under Bed Net					Child Under 5 Slept Under Bed Net				
	Rural	Urban	Non-Poor	Poor	Total	Rural	Urban	Non-Poor	Poor	Total
Symptoms of malaria	.264*** (.066)	.132 (.086)	.017 (.078)	.170** (.069)	.092* (.052)	.257*** (.064)	.115 (.091)	.034 (.084)	.172*** (.066)	.111** (.053)
Control variables	Yes	Yes	Yes	Yes	Yes	Yes	Yes	Yes	Yes	Yes
Observations	2,000	1,235	1,458	1,777	3,235	1,875	1,154	1,357	1,672	3,029
Pseudo *R*^2^	.053	.064	.18	.164	.102	.053	.064	.185	.176	.102
Log pseudolikelihood	−1170.897	−780.558	−921.372	−1023.417	−1974.569	−1170.897	−780.558	−921.372	−1023.417	−1974.569
Specification test	.145	.775	.874	.422	.750	.145	.775	.874	.422	.750
Goodness–of–fit test	.132	.211	.135	.276	.241	.239	.54	.344	.437	.241
Causes of malaria	.017* (.009)	.011 (.012)	.007 (.010)	.014 (.010)	.003 (.007)	.006** (.009)	.012*** (.012)	.009** (.011)	.004 (.010)	.002 (.007)
Control variables	Yes	Yes	Yes	Yes	Yes	Yes	Yes	Yes	Yes	Yes
Observations	2,000	1,235	1,458	1,777	3,235	1,875	1,154	1,357	1,672	3,029
Pseudo *R*^2^	.047	.064	.181			.05	−733.764	.186	.164	
Log pseudolikelihood	−1177.439	−781.339	−921.183	−1025.333	−2014.801	−1094.095	.067	−857.72	−957.946	−2014.801
Specification test	.399	.919	.795	.519	.697	.124	.582	.938	.244	.697
Goodness–of–fit test	.113	.127	.287	.176	.211	.1841	.244	.156	.192	.211
Prevention of malaria	.022** (.009)	.027** (.012)	.015 (.010)	.024** (.010)	.001 (.007)	.038*** (.009)	.025** (.012)	.010 (.011)	.038*** (.010)	.011 (.007)
Control variables	Yes	Yes	Yes	Yes	Yes	Yes	Yes	Yes	Yes	Yes
Observations	2,000	1,235	1,458	1,777	3,235	1,875	1,154	1,357	1,672	3,029
Pseudo *R*^2^	.048	.066	.182	.163	.101	.052	.069	.186	.167	.101
Log pseudolikelihood	−1176.697	−778.992	−920.106	−1023.667	−1975.967	−1092.489	−732.354	−857.198	−955.385	−1975.967
Specification test	.786	.825	.572	.594	.548	.344	.869	.944	.408	.548
Goodness–of–fit test	.222	.331	.476	.633	.218	.203	.239	.163	.219	.218
Overall knowledge	.059*** (.016)	.050** (.021)	.012 (.019)	.046*** (.017)	.015** (.012)	.062*** (.015)	.046** (.022)	.011 (.020)	.049*** (.016)	.020** (.013)
Control variables	Yes	Yes	Yes	Yes	Yes	Yes	Yes	Yes	Yes	Yes
Observations	2,000	1,235	1,458	1,777	3,235	1,875	1,154	1,357	1,672	3,029
Pseudo *R*^2^	.052	.066	.189	.1642	.101	.055	.071	.186	.167	.101
Log pseudolikelihood	−1172.103	−778.951	−920.988	−1022.773	−1975.415	−1088.232	−731.908	−857.769	−955.209	−1975.415
Specification test	0.3	.813	.683	.532	.732	.187	.595	.997	.268	.732
Goodness–of–fit test	.411	.255	.775	.273	.373	.166	.242	.158	.184	.373

Standard errors in parentheses; ***p < .01, **p < .05, *p < .1. The control variables included in the models for the mother sleeping under bed net are her age, education, ownership of insurance, household poverty status, ethnicity, place of residence (geographical location) and regional location (dummies) of the household. In the child’s models, we included the child’s age, sex and the number of children under age 5 in the household as additional control variables. Source: Authors’ computation based on data from MIC 2016.

Although we did not find a significant positive association between women’s knowledge of the causes and prevention of malaria and the likelihood of their sleeping under a bed net, we found statistically significant differences across geographical locations and households’ poverty status. The results generally suggest that improved knowledge of malaria has a statistically higher effect among women who live in rural areas and poor households than among women who live in urban areas and non-poor households.

Regarding the results for the child, a 1% increase in the mother’s knowledge of the symptoms of malaria is associated with an approximately 11% increase in the likelihood of her child under the age of five years sleeping under a bed net, *ceteris paribus*. The extent of association between a woman’s overall knowledge of malaria and the likelihood of her child sleeping under a bed net is about 2%. We further found that the association between a woman’s knowledge of malaria and the likelihood of her child sleeping under a bed net is more significant in poor and rural households than in non-poor and urban households. With reference to the literature, these results corroborate the conclusion of earlier studies (Boulay et al., 2014; Mandal, 2022; Nyunt et al., 2015; Zalisk et al., 2019) that BCC messages are effective in inducing people to take malaria prevention measures.

## Conclusion

Intensive BCC is critical to the efforts of global bodies such as the WHO and its allied institutions to achieve the ambitious objective of eradicating malaria by 2050. However, effective and well-targeted strategies designed to achieve this objective require data and the continuous evaluation of the various BCC policies that have already been rolled out in many developing countries. This study contributes to this effort from a Ghanaian perspective by assessing how women’s exposure to the three mediums of communication (media messages on malaria, participation in community-level education on malaria prevention and control, and exposure to both mediums) affect their knowledge (symptoms, causes and prevention) and overall knowledge of the disease.

The descriptive analysis revealed that knowledge of malaria is higher among women who live in poor and rural households. We further found that the socioeconomic factors that significantly determine women’s exposure to either of the mediums of communication include their level of education, their household’s poverty status and geographical location, and their ethnic affiliations. The treatment effect analysis revealed that women who participated in community-level education, heard/saw media messages on malaria or were exposed to both mediums of communication have significantly higher knowledge of malaria than women who had no access to any of the mediums. The impacts of these strategies on women’s knowledge of the symptoms and prevention of the disease were more significant than their knowledge of the causes thereof. The combined effects of exposure to media messages and participation in community-level education on malaria prevention were relatively higher than the impact of either of them as a single medium of communication.

The analysis further revealed that an improvement in women’s knowledge is significantly associated with malaria prevention measures that they take for themselves and their children under the age of five years. However, the extent of association is statistically higher for knowledge of symptoms and prevention than of causes. The effect is statistically more significant among women living in rural and poor households than among women living in urban and non-poor households. These results suggest that the MOH, the GHS and their partner institutions should adopt an innovative approach which combines the two strategies in intensively educating Ghanaians, and women in particular, on the causes of the disease. Such approaches should take into consideration households’ socioeconomic status and geographical location.

While the findings of this study provide compelling evidence of the effectiveness of the BCC campaign, particularly among those segments of the population that are more vulnerable to malaria, there is a need for more high-quality data, especially as transmission dynamics change. The cross-sectional nature of the data used in this analysis did not permit us to address this gap which has been highlighted in the literature. Future studies could make a considerable further contribution to the literature by attempting to address this gap.

## Note

1. Data for the computation of Figure 1 were extracted from the Demographic and Health Survey (DHS), Multiple Indicator Cluster Survey (MICS) and Malaria Indicator Survey (MIS).

## Appendix

**Table A1.** Correlates of Exposure to the Mediums of Communication.

Variables	Community-Level Education	Media Message	Both Mediums
Basic education (Ref: No education)	.003 (.011)	.037 (.025)	.004 (.014)
Secondary plus	.029** (.012)	.148*** (.024)	.052*** (.017)
Age	.001 (.001)	.003** (.001)	.001 (.001)
Household head (Ref: Other)	.055*** (.018)	.062** (.029)	.061*** (.021)
Other relative	.010 (.010)	.069*** (.021)	.036*** (.013)
Ethnicity – Akan (Ref: Ewe)	−.064*** (.024)	−.006 (.046)	−.099** (.045)
Ethnicity – Ga-Dangme	.017 (.030)	−.068* (.037)	−.047 (.041)
Ethnicity – Guan	−.053** (.027)	−.023 (.052)	−.089** (.044)
Ethnicity – Mole-Dagbani	−.038* (.021)	−.122*** (.033)	−.100*** (.032)
Ethnicity – Grusi	−.019 (.026)	−.174*** (.044)	−.088** (.036)
Ethnicity – Gurma	.013 (.033)	−.112*** (.043)	−.031 (.044)
Ethnicity – Mande	.024 (.042)	−.016 (.077)	−.046 (.048)
Ethnicity – Other	−.016 (.034)	.001 (.052)	−.037 (.058)
Poor household	−.024** (.012)	−.110*** (.022)	−.041** (.017)
Urban household	−.027** (.011)	.036* (.020)	−.012 (.015)
Western region	−.015 (.013)	−.247*** (.042)	−.041* (.022)
Central region	.042** (.019)	.009 (.045)	.065* (.034)
Volta region	.008 (.016)	−.278*** (.042)	−.038* (.023)
Eastern region	−.015 (.013)	−.219*** (.041)	−.040* (.022)
Ashanti region	−.002 (.013)	−.244*** (.039)	−.029 (.022)
Brong–Ahafo region	.031* (.018)	−.152*** (.043)	.015 (.027)
Northern region	.016 (.019)	−.206*** (.047)	.026 (.036)
Upper East region	.221*** (.052)	.209*** (.044)	.479*** (.073)
Upper West region	.202*** (.053)	.018 (.052)	.268*** (.079)
Observations	3,235	3,235	1,943

Standard errors in parentheses; ****p* < .01, ***p* < .05, **p* < .1. Source: Authors’ computation based on data from MIC 2016.

**Table A2.** Extent of Matching Bias Across the Observable Correlates.

Variable	Community-Level Education				Media Message				Both Mediums of Communication			
	Treated	Control	%Bias	*t* test	Treated	Control	%Bias	*t* test	Treated	Control	%Bias	*t* test
Basic education (Ref: None)	.193	.145	12.100	1.310	.193	.198	−1.100	−.160	.195	.128	16.800	1.580
Secondary plus	.493	.517	−4.800	−.490	.493	.583	−18.000	−2.520	.490	.490	.000	.000
Age	30.855	30.266	8.400	.840	30.855	30.314	7.700	1.110	30.550	30.852	−4.300	−.370
Household head (Ref: Other)	.208	.145	17.000	1.680	.208	.137	19.100	2.780	.175	.201	−7.300	−.590
Spouse	.623	.672	−10.100	−1.030	.623	.678	−11.500	−1.620	.691	.698	−1.400	−.130
Ethnicity – Akan (Ref: Ewe)	.005	.005	.000	.000	.005	.057	−34.100	−3.240	.007	.000	4.300	1.000
Ethnicity – Ga-Dangme	.101	.082	6.200	.680	.101	.092	3.000	.440	.034	.047	−4.300	−.590
Ethnicity – Guan	.010	.010	.000	.000	.010	.032	−14.400	−1.790	.013	.007	4.400	.580
Ethnicity – Mole-Dagbani	.353	.367	−3.200	−.310	.353	.243	24.000	3.510	.376	.376	.000	.000
Ethnicity – Grusi	.101	.092	3.800	.330	.101	.036	25.400	4.570	.087	.101	−5.200	−.400
Ethnicity – Gurma	.072	.063	3.400	.390	.072	.049	8.100	1.440	.081	.101	−7.100	−.600
Ethnicity – Mande	.048	.072	−14.200	−1.030	.048	.017	18.100	3.080	.067	.054	7.900	.480
Ethnicity – Other	.019	.010	6.100	.820	.019	.029	−6.400	−.830	.013	.007	4.300	.580
Poor	.594	.599	−1.000	−.100	.594	.411	37.000	5.160	.597	.550	9.500	.820
Urban	.300	.329	−6.100	−.630	.300	.495	−41.200	−5.440	.329	.315	2.800	.250
Western region (Ref: Greater Accra)	.024	.029	−2.200	−.300	.024	.065	−18.300	−2.350	.020	.040	−9.000	−1.010
Central region	.116	.101	4.900	.470	.116	.117	−.400	−.050	.128	.081	15.800	1.330
Volta region	.092	.077	4.800	.530	.092	.064	9.100	1.530	.020	.027	−2.200	−.380
Eastern region	.024	.024	.000	.000	.024	.082	−25.800	−2.990	.020	.013	3.000	.450
Ashanti region	.068	.072	−1.700	−.190	.068	.122	−19.000	−2.320	.060	.087	−9.400	−.880
Brong–Ahafo region	.092	.097	−1.700	−.170	.092	.099	−2.300	−.320	.107	.134	−9.200	−.710
Northern region	.082	.082	.000	.000	.082	.085	−.700	−.120	.107	.101	2.000	.190
Upper East region	.261	.266	−1.300	−.110	.261	.138	32.800	4.790	.336	.336	.000	.000
Upper West region	.198	.227	−8.600	−.720	.198	.071	37.700	6.500	.161	.175	−4.000	−.310

Source: Authors’ computation based on data from MIC 2016.

## References

[bibr1-0193841X231194565] Alkire S. Foster J. (2011). Counting and multidimensional poverty measurement. Journal of Public Economics, 95(7–8), 476–487.

[bibr2-0193841X231194565] Angrist J. D. Kuersteiner G. M. (2011). Causal effects of monetary shocks: Semiparametric conditional independence tests with a multinomial propensity score. The Review of Economics and Statistics, 93(3), 725–747.

[bibr3-0193841X231194565] Apo S. B. Kwankye S. O. Badasu D. M. (2015). Exposure to malaria prevention messages and insecticide treated bednet usage among children under five years in Ghana. European Scientific Journal, 11(18), 290–303.

[bibr4-0193841X231194565] Asmah E. E. Orkoh E. (2017). Self-care knowledge of hypertension prevention and control among women in Contemporary Ghana. American Journal of Health Education, 48(6), 374–381.

[bibr5-0193841X231194565] Biondi-Zoccai G. Romagnoli E. Agostoni P. Capodanno D. Castagno D. D’Ascenzo F. Sangiorgi G. Modena M. G. (2011). Are propensity scores really superior to standard multivariable analysis? Contemporary Clinical Trials, 32(5), 731–740. 21616172 10.1016/j.cct.2011.05.006

[bibr6-0193841X231194565] Boulay M. Lynch M. Koenker H. (2014). Comparing two approaches for estimating the causal effect of behaviour-change communication messages promoting insecticide-treated bed nets: An analysis of the 2010 Zambia malaria indicator survey. Malaria Journal, 13(1), 342. 25174278 10.1186/1475-2875-13-342 PMC4161873

[bibr7-0193841X231194565] Caliendo M. Kopeinig S. (2008). Some practical guidance for the implementation of propensity score matching. Journal of Economic Surveys, 22(1), 31–72.

[bibr8-0193841X231194565] Chen X. Orom H. Hay J. L. Waters E. A. Schofield E. Li Y. Kiviniemi M. T. (2019). Differences in rural and urban health information access and use. The Journal of Rural Health, 35(3), 405–417. 30444935 10.1111/jrh.12335 PMC6522336

[bibr9-0193841X231194565] Clouston S. A. Yukich J. Anglewicz P. (2015). Social inequalities in malaria knowledge, prevention and prevalence among children under 5 years old and women aged 15–49 in Madagascar. Malaria Journal, 14(499), 1–10. 26651615 10.1186/s12936-015-1010-y PMC4676822

[bibr10-0193841X231194565] Doepke M. Zilibotti F. (2017). Parenting with style: Altruism and paternalism in intergenerational preference transmission. Econometrica, 85(5), 1331–1371.

[bibr11-0193841X231194565] Efobi U. Orkoh E. (2017). Assessing youth development in Sub-Saharan Africa with a multidimensional index. PEGNet-Poverty Reduction, Equity and Growth Network, Kiel Institute for the World Economy (IfW Kiel).

[bibr12-0193841X231194565] Ghana Statistical Service (GSS) Ghana Health Service (GHS) ICF . (2017). Ghana malaria indicator survey 2016. GSS, GHS, and ICF. Accra, Ghana, and Rockville.

[bibr13-0193841X231194565] Helinski M. H. Namara G. Koenker H. Kilian A. Hunter G. Acosta A. Scandurra L. Selby R. A. Mulondo K. Fotheringham M. Lynch M. (2015). Impact of a behaviour change communication programme on net durability in eastern Uganda. Malaria Journal, 14(1), 366. 26395330 10.1186/s12936-015-0899-5 PMC4580403

[bibr14-0193841X231194565] Iqbal S. A. Botchway F. Badu K. Wilson N. O. Dei-Adomakoh Y. Dickinson-Copeland C. M. Chinbuah H. Adjei A. A. Wilson M. Stiles J. K. Driss A. (2016). Hematological differences among malaria patients in rural and Urban Ghana. Journal of Tropical Pediatrics, 62(6), 477–486. 27318111 10.1093/tropej/fmw038 PMC5141942

[bibr15-0193841X231194565] Koenker H. Keating J. Alilio M. Acosta A. Lynch M. Nafo-Traore F. (2014). Strategic roles for behaviour change communication in a changing malaria landscape. Malaria Journal, 13(1), 1. 24383426 10.1186/1475-2875-13-1 PMC3882285

[bibr16-0193841X231194565] Lamstein S. Stillman T. Koniz-Booher P. Aakesson A. Collaiezzi B. Williams T. Beall K. Anson M. (2014). Evidence of effective approaches to social and behavior change communication for preventing and reducing stunting and anemia: Report from a systematic literature review. USAID/Strengthening Partnerships, Results, and Innovations in Nutrition Globally (SPRING) Project.

[bibr17-0193841X231194565] Le L. H. Hancer M. (2021). Using social learning theory in examining YouTube viewers’ desire to imitate travel vloggers. Journal of Hospitality and Tourism Technology, 12(3), 512–532.

[bibr18-0193841X231194565] Levy B. R. Myers L. M. (2004). Preventive health behaviors influenced by self-perceptions of aging. Preventive Medicine, 39(3), 625–629. 15313104 10.1016/j.ypmed.2004.02.029

[bibr19-0193841X231194565] Mandal B. (2022). Rural–urban differences in health care access and utilization under the medicaid expansion. Applied Economic Perspectives and Policy, 44(2), 702–721.

[bibr20-0193841X231194565] Ministry of Health (MOH) . (2010). National malaria behaviour change communication strategy. Ministry of Health. https://www.ghanahealthservice.org/downloads/MalariaCommmunicationStrategy.pdf

[bibr21-0193841X231194565] Nabavi R. T. (2012). Bandura’s social learning theory & social cognitive learning theory. Theory of Developmental Psychology, 1(1), 1–24.

[bibr22-0193841X231194565] Nair S. N. Darak S. Parsekar S. S. Menon S. Parchure R. Vijayamma R. Darak T. Nelson H. (2016). Effectiveness of behaviour change communication (bcc) interventions in delivering health messages on antenatal care for improving maternal and child health (mch) indicators in a limited literacy setting: An evidence summary of systematic reviews. Protocol. EPPI-Centre, Social Science Research Unit, UCL Institute of Education, University College London.

[bibr23-0193841X231194565] Nyunt M. H. Aye K. M. Kyaw M. P. Wai K. T. Oo T. Than A. Oo H. W. Phway H. P. Han S. S. Htun T. San K. K. Htun T. (2015). Evaluation of the behaviour change communication and community mobilization activities in Myanmar artemisinin resistance containment zones. Malaria Journal, 14(1), 522. 26697850 10.1186/s12936-015-1047-y PMC4690302

[bibr24-0193841X231194565] Orkoh E. Annim S. K. (2017). Source and use of insecticide treated net and malaria prevalence in Ghana. Social Science Research Network (SSRN).

[bibr25-0193841X231194565] Owusu Adjah E. S. Panayiotou A. G. (2014). Impact of malaria related messages on insecticide-treated net (ITN) use for malaria prevention in Ghana. Malaria Journal, 13(1), 1–7. 24679068 10.1186/1475-2875-13-123 PMC3997841

[bibr26-0193841X231194565] President’s Malaria Initiative (PMI) . (2022). U.S. President’s malaria initiative Ghana malaria operational plan FY 2022. US President’s Malaria Initiative. https://www.pmi.gov.

[bibr27-0193841X231194565] Quakyi I. A. Adjei G. O. Sullivan D. J. Stephens J. K. Laar A. Aubyn V. N. A. Owusu R. Sakyi K. S. Coleman N. Krampa F. D. Vanotoo L. Tuakli J. Bortei B. B. Essuman E. Sorvor F. Boateng I. A. Bart-Plange C. Addison E. A. Winch P. Adjei A. A. (2017). Targeted community based interventions improved malaria management competencies in rural Ghana. Global Health Research and Policy, 2(1), 29. 29202097 10.1186/s41256-017-0048-5 PMC5683319

[bibr28-0193841X231194565] Rosenbaum P. R. (2002). Covariance adjustment in randomized experiments and observational studies. Statistical Science, 17(3), 286–327.

[bibr29-0193841X231194565] Saturni S. Bellini F. Braido F. Paggiaro P. Sanduzzi A. Scichilone N. Santus P. A. Morandi L. Papi A. (2014). Randomized controlled trials and real life studies. Approaches and methodologies: A clinical point of view. Pulmonary Pharmacology & Therapeutics, 27(2), 129–138. 24468677 10.1016/j.pupt.2014.01.005

[bibr30-0193841X231194565] The Health Communication Capacity Collaborative (HC3) . (2017). Malaria SBCC evidence literature review. Johns Hopkins Center for Communication Programs.

[bibr31-0193841X231194565] Tweneboah-Koduah E. Y. Braimah M. Otuo P. N. (2012). Behavioral change communications on malaria prevention in Ghana. Health Marketing Quarterly, 29(2), 130–145. 22676841 10.1080/07359683.2012.678257

[bibr32-0193841X231194565] White H. Sabarwal S. (2014). Quasi-experimental design and methods: Methodological briefs-impact evaluation No. 8. Geneva, Switzerland: United Nations International Children’s Emergency Fund (UNICEF). https://www.unicef-irc.org/publications/753-quasi-experimental-design-and-methods-methodological-briefs-impact-evaluation-no.html.

[bibr33-0193841X231194565] Whittaker M. A. Dean A. J. Chancellor A. (2014). Advocating for malaria elimination-learning from the successes of other infectious disease elimination programmes. Malaria Journal, 13(1), 1–8. 24902848 10.1186/1475-2875-13-221 PMC4057589

[bibr34-0193841X231194565] World Health Organization (WHO) . (2019). Fourth meeting of the WHO strategic advisory group on malaria eradication: Background document for session 4. Geneva, Switzerland: World Health Organization. https://www.who.int/malaria/mpac/mpac-april2019-session4-SAG-malaria-eradication-report.pdf?ua=1.

[bibr35-0193841X231194565] Yilma Z. van Kempen L. de Hoop T. (2012). A perverse ‘net’effect? Health insurance and ex-ante moral hazard in Ghana. Social Science & Medicine, 75(1), 138–147. 22507951 10.1016/j.socscimed.2012.02.035

[bibr36-0193841X231194565] Zalisk K. Herrera S. Inyang U. Mohammed A. B. Uhomoibhi P. Yé Y. (2019). Caregiver exposure to malaria social and behaviour change messages can improve bed net use among children in an endemic country: Secondary analysis of the 2015 Nigeria Malaria Indicator Survey. Malaria Journal, 18(1), 121. 30954073 10.1186/s12936-019-2750-x PMC6451249

[bibr37-0193841X231194565] Zentall T. R. (2022). Mechanisms of copying, social learning, and imitation in animals. Learning and Motivation, 80, 101844. https://www.sciencedirect.com/science/article/abs/pii/S0023969022000649.

